# Speciation of Main Nutrients (N/P/K) in Hydrochars Produced from the Hydrothermal Carbonization of Swine Manure under Different Reaction Temperatures

**DOI:** 10.3390/ma14154114

**Published:** 2021-07-23

**Authors:** Jiangbo Xiong, Shuaiwei Chen, Jiaxin Wang, Yujie Wang, Xiaolin Fang, Huajun Huang

**Affiliations:** 1 School of Land Resources and Environment, Key Laboratory of Agricultural Resource and Ecology in the Poyang Lake Basin of Jiangxi Province, Jiangxi Agricultural University, Nanchang 330045, China; 9707xinlang@sina.com (J.W.); wyj1649757125@126.com (Y.W.); fxl13870703704@163.com (X.F.); 2 School of Forestry, Fujian Agriculture and Forestry University, Fuzhou 350002, China; fjnlchenshuaiwei@163.com

**Keywords:** swine manure, hydrothermal carbonization, hydrochar, nutrients, speciation

## Abstract

Hydrothermal carbonization (HTC) has been proved to be a promising technology for swine manure (SM) treatment. Currently, there is a lack of systematic understanding of the transformation characteristics of nutrient speciation in the HTC of SM. In this study, the speciation of the main nutrients (N/P/K) in SM-derived hydrochar produced at different reaction temperatures (200–280 °C) was investigated. The recovery of P (61.0–67.1%) in hydrochars was significantly higher than that of N (23.0–39.8%) and K (25.5–30.0%), and the increase in reaction temperature promoted the recovery of P and reduced the recovery of N. After the HTC treatment, the percentage of soluble/available P was reduced from 61.6% in raw SM to 4.0–23.9% in hydrochars, while that of moderately labile/slow-release P was improved from 29.2% in raw SM feedstock to 65.5–82.7%. An obvious reduction was also found in the amounts of available N (from 51.3% in raw SM feedstock to 33.0–40.5% in hydrochars). The percentages of slow-release N and residual N in hydrochars produced at 240 °C reached the maximum and minimum values (46.4% and 18.9%), respectively. A total of 49.5–58.3% of K retained in hydrochars was residual (invalid) potassium. From the perspective of the mobility and availability of N, P and K only, it was suggested that the HTC of SM should be carried out at 220–240 °C. Compared with the original SM, it is safer and more effective to use the SM-derived hydrochar as an organic fertilizer.

## 1. Introduction

Livestock production usually generates enormous amounts of manure waste, which demand adequate disposal strategies due to their polluting potentials (e.g., eutrophication, pathogens, antibiotics, and heavy metals) [1,2]. China is the largest pig producer in the world with an average pig rearing amount of 449 million heads per year, accounting for 50% of the total global production [3]. The annual output of livestock manure in China has reached 3.8 billion tons, of which swine manure (SM) accounts for 38.3% [4]. At present, researchers have developed a series of technologies for the treatment/disposal of SM (a wet solid waste), mainly including composting [5,6], anaerobic digestion [7,8], hydrothermal carbonization (HTC) [9,10,11], pyrolysis [12,13], gasification [14,15], and liquefaction [16,17].

HTC, a thermochemical conversion technology, can convert SM into a carbon-rich solid product (hydrochar) under relatively mild temperatures (usually lower than 300 °C) and autogenous pressure for a stipulated residence time [18]. Hydrochar is a kind of multifunctional material, and its application includes use as an adsorbent, for soil remediation/improvement, as fuel, as a carbon-sequestering biochar, and as a substitute for carbon black and activated carbon [19]. During the HTC process, SM is converted with water as a solvent by a series of reactions such as condensation, polymerization, hydrolysis, decarboxylation, dehydration, and aromatization [20]. In other words, no pre-dewatering/drying is needed for the HTC process as required in other thermal treatment processes (e.g., pyrolysis, dry gasification, and combustion), which makes it an economically attractive option for the treatment of SM [19]. Generally speaking, the research on the HTC of SM has been currently carried out regarding the following aspects: (i) the effects of the main process parameters, including reaction temperature/time, solid-liquid ratio, catalyst and heating mode, on the yield/property of hydrochar [21,22,23,24,25]; (ii) the Co-HTC of SM and lignocellulosic biomass [26,27,28]; (iii) the migration and transformation of pollutants (heavy metals and PAHs) existed in raw SM [10,29,30]; (iv) inter-comparison of the properties of hydrochar and biochar (pyrolysis) [9,31,32,33]; and (v) the fate of nutrients contained in raw SM [4,23,34,35].

Nitrogen and phosphorus are the main contributors to the eutrophication of water bodies due to the high release rate of phosphorus and nitrogen in inorganic fertilizers used in agriculture [36]. It has been reported that most of the N (70–80%), P (60–85%) and K (80–90%) in animal diets eventually were transferred to the feces [37]. Thus, SM-derived hydrochar has the potential to be used as organic fertilizer and to solve the problem of nitrogen and phosphorus loss. [23]. Of note, the recycling efficiency of nutrients in hydrochar is greatly influenced by the content and speciation of each nutrient, which ultimately determines the overall quality of hydrochar fertilizer to a large extent [34]. As mentioned above, some studies have been carried out on the fate of nutrients during the HTC of SM, but the focus is primarily on the total content of nutrients retained in hydrochar [23,27,31,32,33]. A few researchers have also paid attention to the contents of nitrate N, ammonium N, available P, and available K in SM-derived hydrochars [4,21,22]. In the research by Huang et al. [34], the transformations of P speciation (including five forms) were explored during the HTC of SM. However, to date, the transformations of N/K speciation during the HTC of SM are still unclear. It is hypothesized that the speciation of N/K would also be changed during the HTC of SM, which is significant for a full understanding of nutrient recovery in SM-derived hydrochars.

The information about the distributions of N/K speciation in SM-derived hydrochars should be further studied. In this work, the transformation characteristics of main nutrients (N, P, and K) speciation were systematically investigated during the HTC of SM. Meanwhile, the recovery rates of N, P, and K were also explored to reflect the overall situation of nutrient recovery. Such information can further guide the selection of process conditions for the HTC of SM from the view of nutrient recovery.

## 2. Materials and Methods

### 2.1. Materials

Fresh SM feedstock was collected from a pig farm near Jiangxi Agricultural University (Nanchang, China). In this preliminary research work, SM feedstock was thoroughly dried to accurately control the amount of solvent (water) used in the HTC process. Specifically, the fresh SM feedstock was firstly air-dried and then transferred to an oven (BPG-9156b, Yiheng, Shanghai, China) for thorough drying (at 105 °C overnight). The dried SM feedstock was crushed, and the samples below 40 mesh were collected. The pretreated SM feedstock was stored in a sealed glass bottle and stored in a desiccator. Some important metals in raw SM feedstock, such as calcium, magnesium, sodium, iron, manganese, and aluminum, were analyzed using an inductively coupled plasma-atomic emission spectrometry (ICP Prodigy XP, Leeman, Hudson, NH, USA) following acid digestion in nitric acid. All measurements were performed thrice and the mean values with standard deviation (SD) are reported in Table 1.

### 2.2. HTC Experiments

The HTC of SM was carried out in a batch reactor (4566, Parr, Moline, IL, USA). In brief, according to the solid–liquid ratio, a certain amount of SM and water were firstly mixed then added to the reactor, and then the reaction was carried out at the desired reaction temperature/time. After the end of the reaction, solid–liquid separation was carried out. Solid-state products were dried, and the yield was calculated. For the detailed HTC process procedure, we referred to the previous work of the authors in [22]. In this work, hydrochar was obtained from the HTC of SM at five different reaction temperatures (200 °C, 220 °C, 240 °C, 260 °C, and 280 °C). The solid–liquid ratio and reaction time were fixed at 0.1 g/mL and 30 min, respectively. It should be emphasized that the reaction time only referred to the reaction duration after the reaction system reached the set reaction temperature, excluding the heating and cooling time.

### 2.3. Speciation Analysis of N/P/K

**Figure 1:**
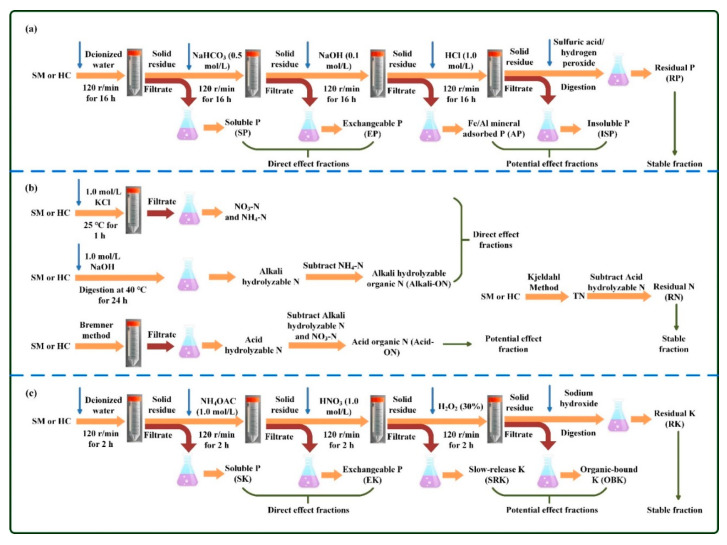
Extraction methods of different chemical forms of phosphorus (**a**), nitrogen (**b**) and potassium (**c**).

Procedures for speciation analysis of nutrient elements (N/P/K) in raw SM and hydrochars are shown in Figure 1. All analysis experiments were carried out three times.

#### 2.3.1. Speciation of Phosphorus (P)

According to the theory of Hedley’s method [34], the speciation of phosphorus (P) includes soluble P (SP), exchangeable P (EP), Fe/Al mineral adsorbed P (AP), insoluble phosphates (ISP), and residual P (RP) (Figure 1a). The contents of SP, EP, AP, and ISP are defined as the amounts of P sequentially extracted by water, NaHCO_3_, NaOH, and HCl, respectively. Specifically, 0.1 g of raw SM or 0.2 g of hydrochar was placed into a 50 mL polypropylene centrifuge tube, which was sequentially extracted by 20 mL extraction solutions, including deionized water, NaHCO_3_ (0.5 mol/L), NaOH (0.1 mol/L) and 1.0 HCl (1.0 mol/L). For each extraction, the mixture oscillated at 120 r/min for 16 h. After the end of each extraction, the mixture was centrifuged and then filtered with a 0.45 μm membrane. The digestion of filtrate was conducted with the aid of persulfate, and then the concentration of P was determined by an ammonium molybdate colorimetric spectrophotometer (DR5000, HACH, Loveland, CO, USA). The residual solids after all extraction were digested with sulfuric acid/hydrogen peroxide solutions, and then the concentration of P in the digestion solution was detected using the method described above, which was defined as RP. The content of total phosphorous (TP) was estimated by adding all of the above speciation of P.

#### 2.3.2. Speciation of Nitrogen (N)

The speciation of N was roughly divided into available, slow-release and residual forms (Figure 1b). The available N includes inorganic available N (mainly NO_3_-N and NH_4_-N) and organic available N. To determine the content of NO_3_-N and NH_4_-N, the raw SM feedstock or hydrochar samples were extracted by 1.0 mol/L KCl at 25 °C for 1 h, and then the corresponding concentrations were detected with the aid of a continuous flow autoanalyzer (AA3, Seal, Norderstedt, Germany). The content of organic available N was defined as the difference between alkali hydrolyzable N and NH_4_-N, also called alkali hydrolyzable organic N (Alkali-ON). The content of alkali hydrolyzable N was determined with the method of NaOH hydrolyzation diffusion (1.0 mol/L, at 40 °C for 24 h) followed by boric acid titration [22]. The content of slow-release N was estimated by subtracting alkali hydrolyzable N and NO_3_-N from acid hydrolyzable N, also called acid organic N (Acid-ON). The content of acid hydrolyzable N was measured according to the Bremner method, which is as follows: firstly, extraction with 6 mol/L hydrochloric acid; secondly, digestion with sulfuric acid and catalyst (K_2_SO_4_-CuSO_4_-Se powder); and lastly, detection with distilled/semi-micro titration [38]. The content of residual N (RN) was defined as the fraction of non-hydrolyzable nitrogen, which was estimated by subtracting acid hydrolyzable N from total nitrogen (TN). The content of TN was determined by the standard Kjeldahl method with the aid of a Kjeldahl apparatus (K1160, Hanon, Jinan, China) [35].

#### 2.3.3. Speciation of Potassium (K)

In general, the speciation of K consists of soluble K (SK), exchangeable K (EK), slow-release K (SRK), organic-bound K (OBK), and residual K (RK) (Figure 1c). The contents of SK, EK, SRK, and OBK were defined as the amounts of K extracted by water, ammonium acetate, nitric acid, and hydrogen peroxide in turn, respectively [39,40,41,42]. In particular, 0.1 g of raw SM feedstock or 0.2 g of hydrochar was placed into a 50 mL polypropylene centrifuge tube, which was sequentially extracted by 20 mL extraction solutions, including deionized water, NH_4_OAC (1.0 mol/L), HNO_3_ (1.0 mol/L), and H_2_O_2_ (30%). For each extraction process, the mixture oscillated at 120 r/min for 2 h. After the end of each extraction process, the mixture was centrifuged and then filtered with a 0.45 μm membrane. The concentration of K in each filtrate was detected by the method of flame photometer (FP 640, AOPU, Shanghai, China). The residual solids after all extraction were digested with sodium hydroxide, and then the concentration of potassium in the digestion solution was detected according to the method mentioned above, which was defined as RK. The content of total potassium (TK) was calculated as the sum of all the above speciation of K.

### 2.4. Definition

The recovery rate (*RR*, %) of nutrients (N, P, and K) in hydrochars was calculated according to Equation (1).

(1) $$RR=\frac{C_{HC}^{TC-i}\times Y_{HC-T}}{C_{SM}^{TC-i}}\times 100$$

where $C_{HC}^{TC-i}$ is the total content of one nutrient in hydrochars (mg/g, dry basis); $C_{SM}^{TC-i}$ is the total content of one nutrient in raw SM (mg/g, dry basis); and *Y_HC-T_* is the yield of hydrochar obtained at one reaction temperature (wt.%), which was described in a previous paper of the authors [22].

## 3. Results and Discussions

### 3.1. Recovery Rate of N/P/K

**Figure 2:**
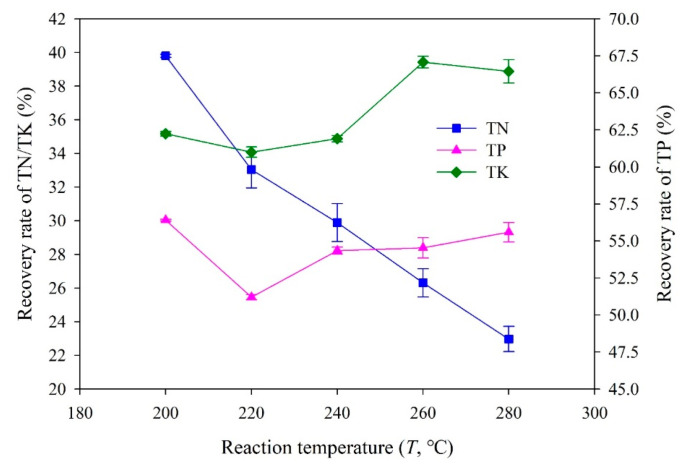
Recovery rate of nutrients in hydrochars obtained at different reaction temperatures.

The recovery rates of three main nutrients (N, P, and K) in hydrochars obtained at different reaction temperatures are shown in Figure 2. A higher recovery rate, 61.0–67.1%, was found for P, while the recovery rates of N and K were relatively low, distributed in the ranges of 23.0–39.8% and 25.5–30.0%, respectively. In addition, the effect of the increase in reaction temperature on the recovery rate of each nutrient element was also not consistent. The recovery rate of P increased overall with the increase in reaction temperature, while that of N showed an opposite trend. The recovery rate of K in hydrochar obtained at 220 °C was the lowest (25.5%). As shown in Table 2, Table 3 and Table 4, the total content of TP in hydrochar increased with the increase in reaction temperature, while that of TN decreased gradually, and that of TK was the lowest at 220 °C. Considering that the yield of hydrochar has the same effect on the recovery rate of different nutrients, the recovery rate results depicted in Figure 2 were reasonable.

Higher reaction temperatures may decrease N recovery to the hydrochar via enhancing deamination reactions, which cause the transportation of N-compounds to the aqueous product (process water) and promotion of the transportation rate of N to the gaseous phase [43]. Regarding P recovery, the increase in reaction temperature improved the formation of metal-associated P species (e.g., calcium, aluminum, magnesium, or iron) and insoluble phosphates, as well as potential physical constraints (e.g., embedment of P species into the char structure) [44,45]. K has a larger water solubility compared to N/P and is a less reactive element. Therefore, on the one hand, the recovery rate of K in hydrochars was relatively low, and on the other hand, the increase in reaction temperature did not bring a significant change to the recovery rate of K [46].

### 3.2. Transformations of P Speciation

**Figure 3:**
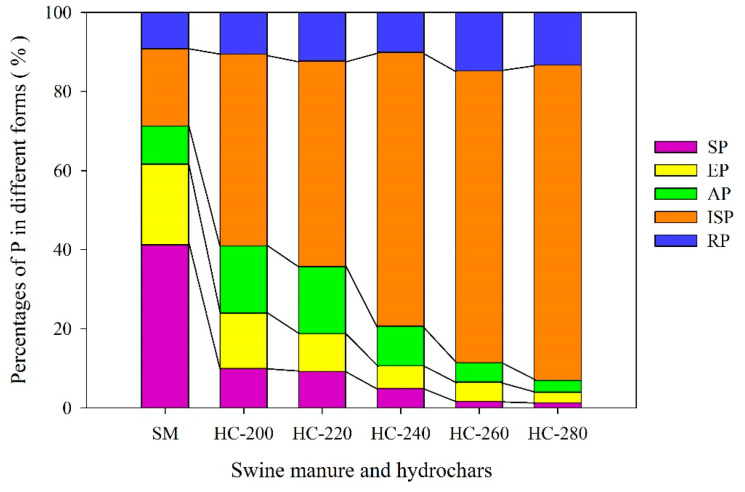
Changes in the forms of P between raw SM and hydrochars obtained at different reaction temperatures.

The content of P present in each form is listed in Table 2. The changes in the proportions of P distributed in different forms between the raw SM feedstock and hydrochars are shown in Figure 3.

#### 3.2.1. H_2_O-Extractable P (SP) and NaHCO_3_-Extractable P (EP)

As seen from Figure 3, about 61.6% of P in the raw SM feedstock can be extracted by H_2_O (SP) and NaHCO_3_ (EP), indicating that P contained in raw SM feedstock was highly mobile. The high percentage of mobile P in raw SM feedstock can largely be attributed to the presence of relatively soluble P species in fresh manure, such as various organic phosphates, soluble phosphates (such as Na and K phosphates) and soluble Ca phosphate species [47,48]. The content of SP in hydrochars reduced sharply with increasing reaction temperature, from 2.73 ± 0.01 mg/g at 200 °C to 0.44 ± 0.04 mg/g at 280 °C, which decreased by 74.4% and 95.9% compared with that in raw SM (10.66 ± 0.07 mg/g), respectively. It was also observed that the proportion of SP decreased from 41.2% for raw SM to less than 10.0% for all hydrochars, which was almost negligible at reaction temperatures higher than 260 °C (less than 2.0%) (Figure 3). These results show that the HTC treatment could greatly reduce the amount of SP in hydrochar, and thus reduce the risk of P loss when it was applied to the soil.

The contents of EP in hydrochars were in the range of 0.92–3.83 mg/g, much lower than those in raw SM (5.27 ± 0.06 mg/g). Furthermore, they continuously decreased with the increase in reaction temperatures and, particularly at 280 °C, they decreased by 82.5% compared with those in the raw SM feedstock. Considering that EP was a kind of biologically available P, it can be concluded that the HTC treatment reduced the bioavailability of P, and the higher the reaction temperature, the lower the bioavailability of P in hydrochars. In addition, as seen in Table 2, the content of SP in hydrochar obtained at 200 °C decreased by 74.4% compared with that in the raw SM feedstock, which was close to the decline range of EP in hydrochar produced at 260 °C (69.4%). This result suggests that the SP was more sensitive to reaction temperature compared to EP. In general, the reduction of SP and EP might be caused by the formation of non-apatite P and apatite P. This inference has been verified in prior studies [24,49,50].

#### 3.2.2. NaOH-Extractable P (AP)

The percentage of NaOH-extractable P (AP) in raw SM feedstock was small (<10%), indicating a low content of Fe/Al mineral adsorbed P species (2.49 ± 0.03 mg/g). At lower HTC reaction temperatures (200–240 °C), the contents of AP in hydrochars were higher than those in raw SM feedstock. However, with the further increase in HTC reaction temperature (260–280 °C), the contents of AP in hydrochars were lower (Table 2). Accordingly, the proportions of AP in hydrochars continuously declined from 17.0% to 3.0% with the increase in reaction temperature (Figure 3). As mentioned, AP was mainly adsorbed on the surface of Fe/Al minerals in manure through chemical adsorption, including inorganic and organic parts, which was a moderately labile P [51]. Thus, the existence of AP was largely related to the concentration of Fe/Al cations. It was deemed that Fe/Al cations can replace K/Na ions to form precipitation at low HTC reaction temperatures, increasing the content of AP in hydrochar, while as the HTC reaction temperature continued to rise, Fe/Al cations may be replaced by calcium ion to form apatite phosphorus or be coated with calcium phosphate, leading to the decrease in AP content [52]. Additionally, the increase in HTC reaction temperature would also accelerate the degradation of organophosphorus, resulting in the decrease in AP in hydrochars to some extent [34].

#### 3.2.3. HCl-Extractable P (ISP) and Residual P (RP)

The HCl-extractable P (ISP) was the apatite P fraction, which is a stable form of P and assumed to be associated with Ca [51]. The percentage of ISP in raw SM feedstock was 19.6%, with the contribution of the insoluble Al and Ca phosphate phases. The content of ISP in hydrochars increased dramatically with the increase in HTC reaction temperature, by 1.6–4.4 times compared with that in the raw SM feedstock (Table 2). Thus, the proportions of ISP in hydrochars were up to 48.5–79.8%, much higher than those in raw SM (19.6%), and increased with increasing reaction temperatures (Figure 3). These results suggest that although P was concentrated in the hydrochars (Figure 1), the amount of insoluble P tended to increase remarkably after the HTC treatment. In other words, the raw SM feedstock was transformed into slow-release phosphate fertilizer by the HTC treatment. The increase in ISP can be explained from the following three aspects: (i) calcium was the main cation in raw SM feedstock, which could provide a prerequisite for the formation of calcium-containing phosphate; (ii) under hydrothermal conditions, calcium and orthophosphate can form calcium-containing phosphate with different solubility and stability, such as amorphous-, crystalline- and hydroxyapatite-calcium; and (iii) the degree of dissolution, reprecipitation and/or crystallization of calcium-containing phosphate were also affected by the reaction temperature. A higher HTC reaction temperature was conducive to the formation of more stable phosphate phases, such as apatite phosphorus [34].

A small amount of P (9.20%) was found in the residual form (RP), possibly due to the incorporation of ore-P in the raw SM feedstock (Figure 3). The contents of RP in hydrochars were higher than those in raw SM, and the percentages of RP in hydrochars fluctuated in the range of 10.13–14.80% with increasing reaction temperatures.

On the whole, the HTC treatment, especially at higher reaction temperatures, could increase the total content of phosphorus in hydrochars. Meanwhile, the percentage of insoluble/stable P in hydrochar was increased, while that of the water-soluble phosphorus and NaHCO_3_-extractable P was reduced. On one hand, the HTC treatment could substantially reduce the mobility of P in hydrochars; on the other hand, for agricultural application, the hydrochars could not only act as an available fertilizer but also as a slow-release fertilizer [52]. From the perspective of the mobility/bioavailability and recovery rate of P, the reasonable HTC reaction temperature for SM might be 240 °C. The hydrochars prepared in this reaction temperature were expected to be effective for the medium and long-term growth of crops and have a lower risk of runoff and leaching losses when applied to soil as an amendment. Too high HTC reaction temperature would make the loss of active phosphorus in hydrochar very problematic.

### 3.3. The Transformations of N Speciation

**Figure 4:**
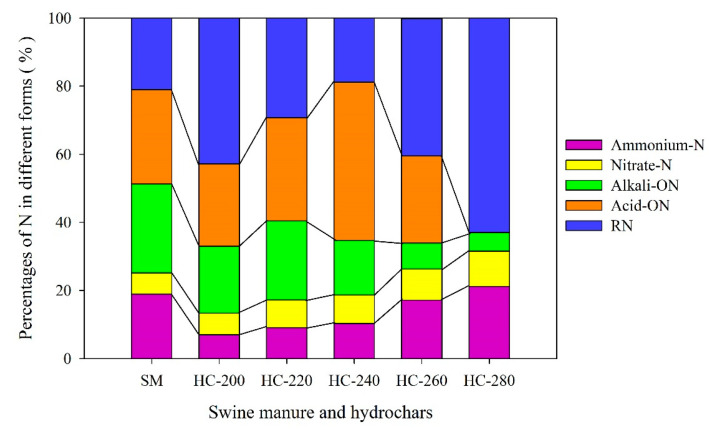
Changes in the forms of N between raw SM and hydrochars obtained at different reaction temperatures.

Table 3 presents the content of N in each form in raw SM feedstock and hydrochar products produced at different reaction temperatures (200–280 °C). Figure 4 depicts the changes in the proportion of N distributed in different forms.

#### 3.3.1. Inorganic Nitrogen (NH_4_-N and NO_3_-N)

NH_4_-N and NO_3_-N belong to inorganic nitrogen, which can be quickly absorbed and utilized by plants, commonly seen as a quick-availability nitrogen fertilizer [4]. As seen in Table 3, the content of inorganic N in raw SM feedstock was 5.95 mg/g, accounting for 25.1% of the total nitrogen (Figure 4). After the HTC treatment, the content of inorganic N in hydrochars decreased significantly by 48.9–74.8% (NH_4_-N) and 23.1–30.6% (NO_3_-N), respectively, compared with those in the raw SM feedstock. This was due to the fact that the inorganic N in the raw SM feedstock was mainly transferred into the aqueous product (process water) during the HTC process in the forms of NH_4_-N and NO_3_-N by hydrolysis [4,53].

It was also found that the effects of reaction temperature on the content of NH_4_-N and NO_3_-N in hydrochars were different. With increasing reaction temperatures, the content of NH_4_-N in hydrochars showed a slightly increasing trend, while that of NO_3_-N changed little, consistent with the research results reported by Kruse et al. [54], who explored the fate of N during the HTC of carrot green, *Chlorella pyrenoidosa*, and straw. Nitrate N was negatively charged and ammonium N was positively charged. The chemical bonding was the main mechanism for the incorporation of NH_4_-N into the hydrochar during HTC, while the sorption (electrostatic adsorption/salts precipitation) was the main mechanism for the recovery of NO_3_-N to the hydrochar [43]. This distinct adsorbate nature might be the main cause of the difference between the changes in the situations of NH_4_-N and NO_3_-N contents.

As noted above, the inorganic nitrogen existing in hydrochars was primarily attributed to the sorption or chemical bonding to the structure of hydrochar. Hydrochar is also an effective adsorbent with a large specific surface area and a large number of charged functional groups (mainly negatively charged) [55,56]. To a certain extent, the increase in reaction temperature can promote the formation of pore structure and surface functional groups in hydrochar. Due to this, it was expected that the proportion of inorganic nitrogen in hydrochar increased from 13.3% (200 °C) to 31.6% (280 °C).

#### 3.3.2. Alkali-Hydrolyzed Organic Nitrogen (Alkali-ON)

Alkali-hydrolyzed organic nitrogen (Alkali-ON) includes simple organic nitrogen, such as amino acids, amides and readily hydrolyzed protein nitrogen, which can be directly absorbed and utilized by plants in the short term. As shown in Table 3, the content of Alkali-ON in raw SM feedstock was up to 6.19 ± 0.04 mg/g, accounting for 26.1% of the total nitrogen (Figure 4). After the HTC treatment, the content of Alkali-ON in hydrochars decreased considerably. Especially at higher reaction temperatures (260–280 °C), the content of Alkali-ON in hydrochars reduced by 85.5–90.6%. These results might be due to the following reactions that occurred during the HTC process: (i) the crude proteins in raw SM were hydrolyzed to soluble polypeptides and amino acids, and then the polypeptides and amino acids were converted to organic acids, amines, ammonia and CO_2_ through deamination and decarboxylation processes; (ii) the polypeptide/amino acids reacted with the reducing sugar or their derivatives through typical Maillard reactions, forming more stable nitrogenous compounds [43,57,58].

With the increase in reaction temperature, the content of Alkali-ON in hydrochars overall showed a decreasing trend. It slightly increased at lower reaction temperatures (200–220 °C), from 3.16 ± 0.06 mg/g at 200 °C up to 3.28 ±0.13mg/g at 220 °C, and slightly decreased at higher reaction temperatures (260–280 °C), from 2.14 ± 0.15 mg/g at 240 °C down to 0.58 ± 0.03 mg/g at 280°C (Table 3). This could be related to the changes in the degradation rate/kinetics of polypeptides and amino acids. The degradation of polypeptides and amino acids was reported mainly in the range of 180–260 °C, and the overall rate would increase at high reaction temperatures and long reaction times. Moreover, different polypeptides/amino acids had distinct kinetics in subcritical water [59].

#### 3.3.3. Acid Hydrolyzed Organic Nitrogen (Acid-ON)

Acid-hydrolyzed organic nitrogen (Acid-ON) refers to the organic nitrogen in the sample, including hexosamine nitrogen and some heterocyclic nitrogen, which could be extracted with hot hydrochloric acid. As it cannot be absorbed directly by plants, it is called slow-release nitrogen. As seen in Table 3, the content of Acid-ON in raw SM feedstock was 6.55 ± 0.26 mg/g, accounting for 27.7% of the total nitrogen (Figure 4). After the HTC treatment, the content of Acid-ON in hydrochar obtained at 240 °C slightly decreased by 5.7% compared with that in the raw SM feedstock. An obvious decrease in the content of Acid-ON in hydrochars produced at other reaction temperatures was found; in particular at 280 °C, it decreased by 99.9%, only 0.01 mg/g.

The content of Acid-ON in hydrochar increased from 3.87 ± 0.11 mg/g at 200 °C to 6.18 ± 0.09 mg/g at 240 °C. This might be due to the continuous hydrolysis of proteins and the conversion of proteins/amino acids to pyrrole-N by cyclization. With the increase in HTC reaction temperature, the cyclization of amine compounds from protein and amino acids, generating pyrrole-N, was enhanced [57]. Thus, the proportion of pyrrole-N in hydrochar continued to increase at 200–240 °C. Significantly, the content of Acid-ON in hydrochar decreased from 6.18 ± 0.09 mg/g at 240 °C to 0.01 mg/g at 280 °C. This might be because the polymerization of pyrrole-N was improved at high reaction temperatures, resulting in the conversion of pyrrole-N into more stable heterocyclic nitrogen forms such as pyridines-N and quaternary-N, which could not be possibly extracted by hydrochloric acid [60,61].

#### 3.3.4. Residual Nitrogen (RN)

Residual nitrogen (RN), which generally exists in heterocyclic form, is bonded to heterocyclic or aromatic rings with a high degree of condensation. As shown in Table 3, the content of residual nitrogen (RN) in hydrochars was firstly reduced and then increased with the increase in HTC reaction temperature (200–280 °C). The lowest content of RN (2.51 mg/g) was found at 240 °C. Notably, the contents of RN in hydrochars produced at 200 °C and 280 °C were 6.87 ± 0.12 mg/g and 6.82 ± 0.15 mg/g, respectively, higher than those in raw SM feedstock (4.99 ± 0.03 mg/g). These results are probably due to the typical Maillard reaction of proteins and amino acids with reducing sugars or their derivatives during the HTC process, which was conducive to the binding of N to aromatic rings, such as pyridine-N and quaternary-N [62]. At a low reaction temperature (200 °C), the Maillard reaction was enhanced due to the abundance of sugar and protein, and the content of quaternary ammonium salt-N in the hydrochar was higher, leading to the increase in RN in hydrochar [57]. When the reaction temperature exceeded 240 °C, more pyridines-N and quaternary-N were formed, resulting in the increase in RN in the hydrochar (240–280 °C) [63].

In summary, after the HTC treatment, the total content of nitrogen retained in the hydrochars significantly reduced compared with that in raw SM feedstock (Figure 2). As depicted in Figure 4, the sum proportion of available nitrogen (Alkali-ON, nitrate N and ammonia N) in hydrochars decreased in comparison with that in raw SM feedstock. The hydrochar prepared at 240 °C contained the highest proportion of slow-release nitrogen (Acid-ON N) and the lowest proportion of residual nitrogen (RN). From the perspective of crop utilization and nutrient loss, moderate available nitrogen and more slow-release nitrogen in hydrochar were expected, which can be obtained by performing the HTC of SM at about 240 °C.

### 3.4. Transformations of K Speciation

**Figure 5:**
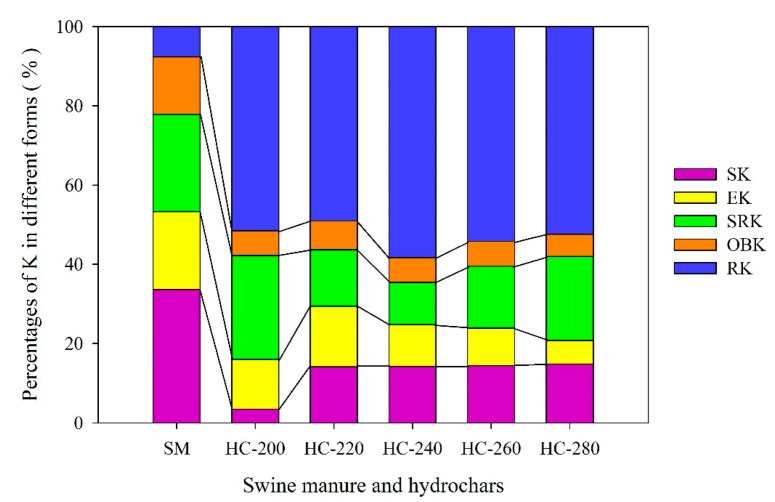
Changes in the forms of K between raw SM and hydrochars obtained at different reaction temperatures.

Table 4 presents the content of each form of K in raw SM feedstock and hydrochar products produced at different reaction temperatures (200–280 °C) through the HTC treatment. Figure 5 depicts the changes in the proportion of different forms of K.

#### 3.4.1. H_2_O-Extractable K (SK) and Ammonium Acetate-Extractable K (EK)

As seen in Table 4, the total content of K in raw SM feedstock reached 14.68 ± 0.72 mg/g, among which the sum content of H_2_O-extractable K (SK) and ammonium acetate-extractable K (EK) was 7.82 ± 0.20 mg/g, accounting for 53.3% of the total potassium (Figure 5). The SK and EK are collectively referred to as available potassium, which can be directly absorbed and utilized by plants. After the HTC treatment, the contents of SK, and EK in hydrochars decreased significantly by 74.3–94.7% and 64.2–82.3%, respectively, compared with those in the raw SM feedstock. Thus, the sum proportion of SK and EK in hydrochars was correspondingly reduced to16.0–29.4% of the total potassium. The proportion of SK in hydrochar increased with the increase in reaction temperature, while that of EK firstly increased and then decreased, with a peak value (15.3%) at 220 °C. This might be because the available K was mainly adsorbed on the surface of hydrochar by physical adsorption. In general, the surface pores of hydrochars would increase with the rise of reaction temperature. On the one hand, with the increase in reaction temperature, the hydrochar matrix had strong adsorption to K, also promoting the formation of a more stable K [49].

#### 3.4.2. HNO_3_-Extractable Potassium (SRK) and Organic-Bound Potassium (OBK)

The contents of HNO_3_-extractable potassium (SRK) and organic-bound potassium (OBK) in raw SM feedstock were 3.61 ± 0.32 mg/g and 2.13 ± 0.13 mg/g, accounting for 24.6% and 14.5% of the total potassium, respectively. Both SRK and OBK can be gradually converted to effective K (available for plant growth). Compared to the raw SM feedstock, the contents of SRK and OBK in hydrochars decreased clearly by 45.9–73.4% and 77.0–77.9%, respectively. A total of 10.7–26.2% of K in hydrochars belonged to SRK and the percentage of OBK in hydrochars was reduced to less than 10%. The proportion of SRK in hydrochar firstly decreased and then increased with increasing reaction temperatures, with the lowest value at 240 °C (10.7%, Figure 5). The contents of SRK in hydrochars obtained at 200 °C and 280 °C were higher, i.e., 1.97 ± 0.08 mg/g and 1.82 ± 0.11 mg/g, respectively. The reason for the former may be due to incomplete hydrolysis of SM, while the latter may be mainly due to the enrichment effect of hydrochar (i.e., reduction of easily degradable substances in SM indirectly increased the content of SRK in hydrochars). As expected, the percentage of OBK in hydrochars fluctuated in a narrow range of 5.6–7.3% with the increase in reaction temperatures, since the content of OBK in hydrochars obtained at different reaction temperatures changed little, possibly because the organic-bound potassium was almost completely degraded at 200 °C.

#### 3.4.3. Residual Potassium (RK)

A small amount of residual potassium (RK, 7.6% of total K), which was also named invalid potassium (not available for plant growth), was contained in the raw SM feedstock (Figure 5). Of note, the content of RK in hydrochars increased significantly (by 2.46–3.04 times compared with that in the raw SM feedstock; Table 4). The proportions of RK in hydrochars rose to 49.5–58.3% (Figure 5). The reason for the above results may be that the increase in reaction temperatures improved the formation of crystalline potassium.

In general terms, after the HTC treatment, the content of total potassium in hydrochars decreased significantly, which decreased by 41.6–54.2% compared with that in the raw SM. Most of the potassium in raw SM feedstock was converted into invalid potassium (RK) in hydrochars after the HTC treatment. In other words, only part of K in hydrochars could be used by crops. To maximize the utilization of K, the HTC of SM was suitably fixed at about 220 °C.

### 3.5. Prospects

The HTC treatment of SM can promote the enrichment of phosphorus in hydrochars, and, at the same time, passivate nitrogen, phosphorus, and potassium to a certain extent. Thus, compared to the direct use of raw SM, the application of SM-derived hydrochar would resolve the nitrogen and phosphorus run-off problems by reducing the mobile phosphorus and nitrogen forms to equilibrate the plant uptake and soil retention capacity [64]. According to the content and proportion of available and slow-release nutrients (N/P/K) retained in hydrochar, it was suggested that the appropriate reaction temperature for the HTC of SM should be approximately 220–240 °C. It should be noted that this suggestion was only based on the chemical speciation of N/P/K nutrients in hydrochars, ignoring the soil environment, crop species and other factors. The ultimate fertility of hydrochars depended upon the interaction among hydrochars, plants, and soils.

## 4. Conclusions

After HTC treatment, phosphorus was enriched in hydrochars, while the contents of nitrogen and potassium were decreased. Of importance, the above three nutrients were all passivated to a certain extent. That is, the contents of directly soluble or active N/P/K were significantly reduced, while the contents of slow-release or stable N/P/K were increased. In this way, it can greatly alleviate environmental pollution caused by the loss of nutrients when SM is directly used in agriculture. The increase in reaction temperatures (200–280 °C) would promote the recovery rate of phosphorus (from 61.0% to 67.1%) and reduce the recovery rate of nitrogen (from 39.8% to 23.0%). From the perspective of the mobility, availability, and recovery rate of N, P, and K only, it was suggested that the HTC of SM should be carried out at about 220–240 °C. There is no doubt that these results will provide an important reference for the utilization of SM-derived hydrochars as organic fertilizers.

## Figures and Tables

**Table 1:** Some important metal contents in raw SM feedstocks.

Items	Concentrations (mg/g, Dry Basis)					
	Ca	Mg	Fe	Mn	Al	Na
SM	17.16 ± 0.25	7.88 ± 0.08	6.93 ± 0.51	0.63 ± 0.02	5.96 ± 0.33	4.52 ± 0.34

**Table 2:** Different forms of phosphorus in raw SM and hydrochars obtained at different reaction temperatures (mg/g, dry basis).

Sample	SP	EP	AP	ISP	RP	∑ (TP)
SM	10.66 ± 0.07	5.27 ± 0.06	2.49 ± 0.03	5.06 ± 0.04	2.38 ± 0.15	25.86 ± 0.35
HC-200	2.73 ± 0.01	3.83 ± 0.02	4.67 ± 0.25	13.29 ± 0.08	2.89 ± 0.07	27.41 ± 0.44
HC-220	2.62 ± 0.01	2.70 ± 0.01	4.83 ± 0.13	14.77 ± 0.02	3.49 ± 0.05	28.41 ± 0.22
HC-240	1.47 ± 0.02	1.74 ± 0.03	3.01 ± 0.05	20.83 ± 0.04	3.05 ± 0.16	30.10 ± 0.30
HC-260	0.52 ± 0.02	1.61 ± 0.01	1.59 ± 0.02	24.08 ± 0.45	4.83 ± 0.13	32.63 ± 0.63
HC-280	0.44 ± 0.04	0.92 ± 0.01	1.01 ± 0.02	27.29 ± 0.52	4.56 ± 0.28	34.22 ± 0.87

**Table 3:** Different forms of nitrogen in raw SM and hydrochars obtained at different reaction temperatures (mg/g, dry basis).

Sample	NH_4_-N	NO_3_-N	Alkali-ON	Acid-ON	RN	∑ (TN)
SM	4.48 ± 0.03	1.47 ± 0.06	6.19 ± 0.04	6.55 ± 0.26	4.99 ± 0.03	23.68 ± 1.04
HC-200	1.12 ± 0.02	1.02 ± 0.04	3.16 ± 0.06	3.87 ± 0.11	6.87 ± 0.12	16.05 ± 0.67
HC-220	1.28 ± 0.02	1.14 ± 0.03	3.28 ± 0.13	4.27 ± 0.16	4.12 ± 0.05	14.09 ± 0.16
HC-240	1.37 ± 0.11	1.11 ± 0.07	2.14 ± 0.15	6.18 ± 0.09	2.51 ± 0.04	13.31 ± 0.08
HC-260	2.01 ± 0.08	1.07 ± 0.12	0.90 ± 0.04	2.99 ± 0.07	4.73 ± 0.06	11.72 ± 0.14
HC-280	2.29 ± 0.07	1.13 ± 0.05	0.58 ± 0.03	0.01 ± 0.05	6.82 ± 0.15	10.83 ± 0.12

**Table 4:** Different forms of potassium in raw SM and hydrochars obtained at different reaction temperatures (mg/g, dry basis).

Sample	SK	EK	SRK	OBK	RK	∑ (TK)
SM	4.94 ± 0.06	2.88 ± 0.14	3.61 ± 0.32	2.13 ± 0.13	1.12 ± 0.07	14.68 ± 0.72
HC-200	0.26 ± 0.02	0.94 ± 0.05	1.97 ± 0.08	0.47 ± 0.01	3.87 ± 0.23	7.51 ± 0.38
HC-220	0.95 ± 0.03	1.03 ± 0.03	0.96 ± 0.05	0.49 ± 0.02	3.33 ± 0.16	6.73 ± 0.29
HC-240	1.11 ± 0.01	0.82 ± 0.01	0.83 ± 0.04	0.49 ± 0.01	4.54 ± 0.37	7.79 ± 0.44
HC-260	1.13 ± 0.07	0.74 ± 0.02	1.23 ± 0.02	0.49 ± 0.02	4.25 ± 0.42	7.84 ± 0.55
HC-280	1.27 ± 0.06	0.51 ± 0.01	1.82 ± 0.11	0.48 ± 0.03	4.49 ± 0.38	8.57 ± 0.59

## Data Availability

Exclude this statement.
